# Supplementary material for: Pre-migration socioeconomic status and post-migration health satisfaction among Syrian refugees in Germany: A cross-sectional analysis
Source: PLoS Med. 2020 Mar 31;17(3):e1003093. doi: 10.1371/journal.pmed.1003093 (PMC7108713; doi:10.1371/journal.pmed.1003093)
Supplement: S1 Table — (DOCX) [file pmed.1003093.s001.docx]

S1 Table Correlation between health indicators

|  | HS | SRH | MH | LS |
| --- | --- | --- | --- | --- |
| Health satisfaction (HS) | 1 |  |  |  |
| Self-rated health (SRH) | 0.8034** | 1 |  |  |
| Mental health (MH) | 0.2862** | 0.332** | 1 |  |
| Life satisfaction (LS) | 0.305** | 0.2637** | 0.3332** | 1 |
| Notes: All health indicators refer to T1 (after migration). Spearman correlations use all available observations that differ due to missing values. ** p < 0.05. | | | | |
