# Supplementary material for: Pre-migration socioeconomic status and post-migration health satisfaction among Syrian refugees in Germany: A cross-sectional analysis
Source: PLoS Med. 2020 Mar 31;17(3):e1003093. doi: 10.1371/journal.pmed.1003093 (PMC7108713; doi:10.1371/journal.pmed.1003093)
Supplement: S2 Table — SES, socioeconomic status. (DOCX) [file pmed.1003093.s002.docx]

S2 Table. Descriptive statistics by Socioeconomic Status

|  | Well below average | Below average | Average | Above average | Well above average |
| --- | --- | --- | --- | --- | --- |
| *Individual measures* | | | | | |
| **Sex** |  |  |  |  |  |
| Female | 37 (36%) | 92 (34%) | 407(39%) | 178 (33%) | 72 (35%) |
| Male | 67 (64%) | 180 (66%) | 628 (61%) | 357 (67%) | 136 (65%) |
| Age (years, mean) | 34 (8.9, 19-61) | 34 (10.5, 18-64) | 33.9 (10.6, 18-75) | 35.5 (10.9, 18-72) | 36.5 (11, 18-65) |
| **Marital status** |  |  |  |  |  |
| Single | 22 (21%) | 80 (30%) | 279 (27%) | 138 (26%) | 43 (21%) |
| Married | 71 (69%) | 187 (69%) | 729 (71%) | 375 (70%) | 159 (77%) |
| Divorced | 7 (7%) | 2 (1%) | 10 (1%) | 11 (2%) | 5 (2%) |
| Widowed | 3 (3%) | 2 (1%) | 14 (1%) | 11 (2%) | 0 (0%) |
| Number of children | 2.2 (2.2, 0-9) | 2 (2.1, 0-9) | 2 (2.2, 0-19) | 2.2 (2.2, 0-13) | 2.3 (2.3, 0-12) |
| *Pre-crisis measures/ Syria (T0)* | | | | | |
| **Educational attainment at T0** | | | | | |
| Left with no qualifications | 30 (29%) | 85 (31%) | 247 (24%) | 111 (21%) | 38 (18%) |
| Middle school | 18 (17%) | 59 (22%) | 219 (21%) | 114 (21%) | 41 (20%) |
| Further practical-based | 7 (7%) | 21 (8%) | 91 (9%) | 49 (9%) | 20 (10%) |
| Further general-based | 26 (25%) | 52 (19%) | 285 (28%) | 184 (34%) | 79 (38%) |
| Other certificate | 4 (4%) | 7 (3%) | 26 (3%) | 16 (3%) | 8 (4%) |
| Educational details N/A | 19 (18%) | 48 (18%) | 167 (16%) | 61 (11%) | 22 (11%) |
| **Income in T0** |  |  |  |  |  |
| 1st quartile | 33 (32%) | 61 (22%) | 158 (15%) | 40 (7%) | 13 (6%) |
| 2nd quartile | 14 (13%) | 50 (18%) | 175 (17%) | 54 (10%) | 11 (5%) |
| 3rd quartile | 13 (13%) | 32 (12%) | 144 (14%) | 109 (20%) | 20 (10%) |
| 4th quartile | 4 (4%) | 14 (5%) | 88 (9%) | 111 (21%) | 78 (38%) |
| Income N/A | 40 (38%) | 115 (42%) | 470 (45%) | 221 (41%) | 86 (41%) |
| Health satisfaction at T0 (mean) | 6.7 (3.5, 0-10) | 7.8 (2.5, 0-10) | 8.4 (2.1, 0-10) | 9 (1.6, 0-10) | 9.3 (1.8, 0-10) |
| Life satisfaction at T0 (mean) | 4.8 (3.3, 0-10) | 5.8 (2.7, 0-10) | 7.6 (2.3, 0-10) | 8.6 (1.9, 0-10) | 9.1 (1.8, 0-10) |
| Subjective SES at T0 (mean) | 0 (0, 0-0) | 1 (0, 1-1) | 2 (0, 2-2) | 3 (0, 3-3) | 4 (0, 4-4) |
| *Post-crisis measures/ Germany (T1)* | | | | | |
| Health satisfaction at T1 (mean) | 7.5 (2.9, 0-10) | 7.8 (2.7, 0-10) | 8 (2.3, 0-10) | 8 (2.3, 0-10) | 8.1 (2.5, 0-10) |
| Life satisfaction at T1 (mean) | 6.9 (2.4, 0-10) | 7 (2.3, 0-10) | 7.3 (2.2, 0-10) | 7.4 (2.1, 0-10) | 7 (2.5, 0-10) |
| Mental health* at T1 (mean) | 8.7 (3, 0-12) | 8.6 (2.7, 0-12) | 9.2 (2.6, 0-12) | 9.1 (2.5, 0-12) | 8.9 (3.2, 0-12) |
| Worries about health* at T1 (mean) | 1.4 (0.7, 0-2) | 1.5 (0.7, 0-2) | 1.5 (0.7, 0-2) | 1.5 (0.7, 0-2) | 1.6 (0.6, 0-2) |
| Self-rated health at T1 (mean) | 3.8 (1.2, 1-5) | 3.9 (1.2, 1-5) | 4 (1, 1-5) | 4 (1.1, 1-5) | 4 (1.1, 1-5) |
| Unemployed at T1 | 101 (97%) | 258 (95%) | 946 (91%) | 474 (89%) | 181 (87%) |
| *Migration experience* | | | | | |
| Number of negative experiences (mean) | 0.7 (1.5, 0-7) | 0.6 (1.1, 0-6) | 0.6 (1.1, 0-6) | 0.7 (1.2, 0-6) | 0.9 (1.2, 0-6) |
| **Duration of migration** |  |  |  |  |  |
| less than 1 year | 55 (53%) | 132 (49%) | 583 (56%) | 257 (48%) | 99 (48%) |
| 1 year | 17 (16%) | 41 (15%) | 160 (15%) | 102 (19%) | 47 (23%) |
| 2 years | 11 (11%) | 37 (14%) | 118 (11%) | 71 (13%) | 26 (13%) |
| 3 years | 9 (9%) | 16 (6%) | 65 (6%) | 35 (7%) | 17 (8%) |
| 4 year or more | 7 (7%) | 20 (7%) | 42 (4%) | 38 (7%) | 14 (7%) |
| Duration N/A | 5 (5%) | 26 (10%) | 67 (6%) | 32 (6%) | 5 (2%) |
| Feeling welcome | 3.7 (0.6, 1-4) | 3.6 (0.7, 0-4) | 3.6 (0.8, 0-4) | 3.5 (0.9, 0-4) | 3.6 (0.8, 0-4) |
| **Year of arrival** |  |  |  |  |  |
| 2013 | 5 (5%) | 15 (6%) | 40 (4%) | 29 (5%) | 19 (9%) |
| 2014 | 21 (20%) | 51 (19%) | 222 (21%) | 172 (32%) | 54 (26%) |
| 2015 | 72 (69%) | 189 (69%) | 721 (70%) | 300 (56%) | 129 (62%) |
| 2016 | 6 (6%) | 17 (6%) | 52 (5%) | 34 (6%) | 6 (3%) |
| Max. n per column | 104 | 272 | 1035 | 535 | 208 |
| Notes: Cell numbers are absolute frequencies (%) or means (standard deviation, range). SES = socioeconomic status, N/A = not available; Mental health is the sum score of the 4 PHQ-4 items. Descriptive statistics are based on data for the specific cells. Variables with a * were inverted so that higher numeric values correspond to better health in all measures. | | | | | |
