# Supplementary material for: Pre-migration socioeconomic status and post-migration health satisfaction among Syrian refugees in Germany: A cross-sectional analysis
Source: PLoS Med. 2020 Mar 31;17(3):e1003093. doi: 10.1371/journal.pmed.1003093 (PMC7108713; doi:10.1371/journal.pmed.1003093)
Supplement: S3 Table — SES, socioeconomic status. (DOCX) [file pmed.1003093.s003.docx]

S3 Table Mental health, SES and migration experience

|  | (1) | (2) |
| --- | --- | --- |
|  | Mental | SES |
| Male | 0.3683*** | 0.0186 |
|  | [0.13,0.61] | [-0.06,0.10] |
| Age | 0.0300 | -0.0062 |
|  | [-0.03,0.09] | [-0.03,0.01] |
| Age² | -0.0006 | 0.0002 |
|  | [-0.00,0.00] | [-0.00,0.00] |
| Financial fraud or financial exploitation | -0.5730*** | 0.1559*** |
|  | [-0.91,-0.23] | [0.04,0.27] |
| Sexual harassment | 0.4453 | -0.4597* |
|  | [-0.92,1.81] | [-0.99,0.07] |
| Physical attacks | -0.8251*** | -0.0657 |
|  | [-1.36,-0.29] | [-0.25,0.12] |
| Shipwreck | -0.3874* | -0.0928 |
|  | [-0.84,0.07] | [-0.26,0.07] |
| Robbery | 0.1395 | 0.0948 |
|  | [-0.32,0.60] | [-0.06,0.25] |
| Blackmail | -0.1948 | 0.1655** |
|  | [-0.63,0.24] | [0.02,0.31] |
| Imprisonment | 0.1520 | -0.0483 |
|  | [-0.19,0.50] | [-0.18,0.08] |
| Intercept | 8.6660*** | 2.1583*** |
|  | [7.55,9.78] | [1.79,2.53] |
| *N* | 2055 | 2153 |
| adj. *R*^2^ | 0.02 | 0.01 |
| Notes: Results based on OLS. 95% CIs based on heteroskedastic robust standard errors in brackets. * p < 0.1, ** p < 0.05, *** p < 0.01. | | |
