# Supplementary material for: Pre-migration socioeconomic status and post-migration health satisfaction among Syrian refugees in Germany: A cross-sectional analysis
Source: PLoS Med. 2020 Mar 31;17(3):e1003093. doi: 10.1371/journal.pmed.1003093 (PMC7108713; doi:10.1371/journal.pmed.1003093)
Supplement: S4 Table — (DOCX) [file pmed.1003093.s004.docx]

S4 Table Replication of Table 2 using an ordered logit model

|  | (1) | (2) | (3) | (4) | (5) |
| --- | --- | --- | --- | --- | --- |
|  | Health Satisfaction | Self-rated Health | Mental  Health | Health Worries | Life Satisfaction |
| *Panel A: Covariates: sex, age* | | | | | |
|  | | | | | |
| SES in T0 | 0.12*** | 0.13*** | 0.09** | 0.19*** | 0.05 |
|  | [0.03,0.21] | [0.04,0.22] | [0.01,0.18] | [0.09,0.28] | [-0.03,0.13] |
| *N* | 2152 | 2152 | 2010 | 2141 | 2140 |
| *Panel B: Covariates: sociodemographics* | | | | | |
|  | | | | | |
| SES in T0 | 0.12** | 0.11** | 0.11** | 0.20*** | 0.09** |
|  | [0.03,0.21] | [0.01,0.21] | [0.02,0.21] | [0.09,0.30] | [0.00,0.19] |
| *N* | 2082 | 2082 | 1945 | 2071 | 2070 |
| *Panel C: Covariates: sociodemographics + migration experience* | | | | | |
|  | | | | | |
| SES in T0 | 0.13*** | 0.11** | 0.12** | 0.20*** | 0.10** |
|  | [0.03,0.22] | [0.01,0.21] | [0.03,0.22] | [0.10,0.31] | [0.00,0.19] |
| *N* | 2082 | 2082 | 1945 | 2071 | 2070 |
| *Panel D: Covariates: sociodemographics + migration experience + experience in Germany* | | | | | |
|  | | | | | |
| SES in T0 | 0.13*** | 0.11** | 0.12** | 0.21*** | 0.09* |
|  | [0.03,0.23] | [0.01,0.21] | [0.02,0.21] | [0.10,0.32] | [-0.00,0.18] |
| *N* | 2065 | 2065 | 1936 | 2058 | 2057 |
| Notes: Results based on an ordered logit model. Covariates included in Panel A: sex, age, age². Added in Panel B: marital status, income at T0, educational attainment at T0, number of children, Syrian birth region dummies. Panel C: neg. migration experience, duration of migration. Panel D: employment status at T1, feeling of welcome, year of arrival. N = number of individuals. 95% CIs based on heteroskedastic robust standard errors in brackets. * p < 0.1, ** p < 0.05, *** p < 0.01. | | | | | |
