# Supplementary material for: Pre-migration socioeconomic status and post-migration health satisfaction among Syrian refugees in Germany: A cross-sectional analysis
Source: PLoS Med. 2020 Mar 31;17(3):e1003093. doi: 10.1371/journal.pmed.1003093 (PMC7108713; doi:10.1371/journal.pmed.1003093)
Supplement: S5 Table — (DOCX) [file pmed.1003093.s005.docx]

S5 Table Replication of Table 3 presenting detailed results on covariates

|  | (1) | (2) | (3) | (4) | (5) |
| --- | --- | --- | --- | --- | --- |
|  | OLS | OLS | OLS | OLS | FE |
| SES in T0 | 0.64*** | 0.63^***^ | 0.63*** | 0.61*** | 0.00 |
|  | [0.53,0.75] | [0.51,0.74] | [0.52,0.74] | [0.50,0.73] | [0.00,0.00] |
| T1 | 0.50*** | 0.53^***^ | 0.53*** | 0.52*** | 0.49*** |
|  | [0.17,0.82] | [0.19,0.86] | [0.19,0.86] | [0.18,0.85] | [0.16,0.82] |
| SES x T1 | -0.48*** | -0.49^***^ | -0.49*** | -0.49*** | -0.47*** |
|  | [-0.61,-0.35] | [-0.62,-0.36] | [-0.62,-0.36] | [-0.62,-0.35] | [-0.60,-0.34] |
| Male | 0.07 | 0.01 | 0.02 | -0.03 |  |
|  | [-0.09,0.23] | [-0.19,0.20] | [-0.17,0.21] | [-0.22,0.17] |  |
| Age | -0.01 | -0.02 | -0.02 | -0.03 |  |
|  | [-0.05,0.03] | [-0.07,0.03] | [-0.07,0.03] | [-0.08,0.02] |  |
| Age² | -0.00* | -0.00 | -0.00 | -0.00 |  |
|  | [-0.00,0.00] | [-0.00,0.00] | [-0.00,0.00] | [-0.00,0.00] |  |
| Marital status | *Single as reference* | | | | |
| Married |  | 0.02 | 0.02 | 0.06 |  |
|  |  | [-0.25,0.29] | [-0.25,0.29] | [-0.21,0.33] |  |
| Divorced |  | -0.58^*^ | -0.53 | -0.48 |  |
|  |  | [-1.26,0.10] | [-1.20,0.14] | [-1.14,0.19] |  |
| Widowed |  | -0.10 | -0.07 | -0.23 |  |
|  |  | [-0.84,0.64] | [-0.81,0.67] | [-0.99,0.52] |  |
| Income in T0 | *1^st^ quartile as reference* | | | | |
| 2^nd^ quartile |  | 0.16 | 0.15 | 0.14 |  |
|  |  | [-0.14,0.47] | [-0.15,0.45] | [-0.16,0.45] |  |
| 3^rd^ quartile |  | 0.04 | 0.05 | 0.06 |  |
|  |  | [-0.26,0.34] | [-0.25,0.35] | [-0.25,0.36] |  |
| 4^th^ quartile |  | 0.01 | 0.03 | 0.05 |  |
|  |  | [-0.30,0.32] | [-0.29,0.34] | [-0.27,0.36] |  |
| Income N/A |  | -0.01 | -0.03 | -0.05 |  |
|  |  | [-0.26,0.25] | [-0.29,0.22] | [-0.30,0.21] |  |
| Education | *No qualification as reference* | | | | |
|  |  |  |  |  |  |
| Middle school |  | 0.15 | 0.17 | 0.16 |  |
|  |  | [-0.09,0.38] | [-0.07,0.40] | [-0.08,0.39] |  |
| Practical-based further education |  | 0.28^*^ | 0.30^*^ | 0.28^*^ |  |
|  |  | [-0.02,0.59] | [-0.01,0.61] | [-0.03,0.58] |  |
| General-based further education |  | 0.32^***^ | 0.32^***^ | 0.34^***^ |  |
|  |  | [0.10,0.53] | [0.10,0.54] | [0.12,0.55] |  |
| Certificate from a different school |  | 0.05 | 0.08 | 0.09 |  |
|  |  | [-0.45,0.55] | [-0.42,0.58] | [-0.39,0.58] |  |
| Education N/A |  | -0.18 | -0.18 | -0.16 |  |
|  |  | [-0.48,0.12] | [-0.48,0.12] | [-0.46,0.14] |  |
| Number of children |  | 0.15 | 0.17 | 0.16 |  |
|  |  | [-0.09,0.38] | [-0.07,0.40] | [-0.08,0.39] |  |
| Negative migration experience (count) |  |  | -0.13^***^ | -0.14^***^ |  |
|  |  |  | [-0.20,-0.05] | [-0.21,-0.06] |  |
| Duration of migration | *Less than 1 year as reference* | | | | |
| 1 year |  |  | -0.02 | -0.02 |  |
|  |  |  | [-0.25,0.20] | [-0.24,0.20] |  |
| 2 years |  |  | 0.20^*^ | 0.17 |  |
|  |  |  | [-0.03,0.44] | [-0.08,0.41] |  |
| 3 years |  |  | -0.10 | -0.14 |  |
|  |  |  | [-0.41,0.21] | [-0.45,0.18] |  |
| 4 years or more |  |  | -0.04 | -0.05 |  |
|  |  |  | [-0.39,0.31] | [-0.40,0.30] |  |
| Duration N/A |  |  | -0.20 | -0.18 |  |
|  |  |  | [-0.56,0.15] | [-0.53,0.17] |  |
| Current employment status | *Full-time as reference* | | | | |
| part-time |  |  |  | -0.12 |  |
|  |  |  |  | [-0.73,0.49] |  |
| minimal or irregular |  |  |  | -0.67 |  |
|  |  |  |  | [-1.94,0.60] |  |
| in-company training |  |  |  | 0.19 |  |
|  |  |  |  | [-0.28,0.66] |  |
| not working |  |  |  | -0.33^*^ |  |
|  |  |  |  | [-0.69,0.02] |  |
| Internship |  |  |  | 0.31 |  |
|  |  |  |  | [-0.37,0.99] |  |
| Feeling welcome upon arrival |  |  |  | 0.11^**^ |  |
|  |  |  |  | [0.02,0.20] |  |
| Intercept | 8.02^***^ | 8.17^***^ | 8.25^***^ | 8.30^***^ | 8.49^***^ |
|  | [7.27,8.77] | [7.27,9.06] | [7.37,9.14] | [7.22,9.39] | [8.44,8.55] |
| Syrian regional dummies | No | Yes | Yes | Yes | No |
| Year of arrival | No | No | No | Yes | No |
| *Number of observations* | 4302 | 4162 | 4162 | 4128 | 4304 |
| adj. *R*^2^ | 0.09 | 0.10 | 0.10 | 0.11 | 0.07 |
| Notes: Dependent variable for all regression: health satisfaction. Results in column 1-4 based on OLS. Column 5 based on within-estimator accounting for individual fixed-effects. 95% CIs based on heteroskedastic robust standard errors clustered on the individuum in brackets. * p < 0.1, ** p < 0.05, *** p < 0.01. | | | | | |
