# Supplementary material for: Pre-migration socioeconomic status and post-migration health satisfaction among Syrian refugees in Germany: A cross-sectional analysis
Source: PLoS Med. 2020 Mar 31;17(3):e1003093. doi: 10.1371/journal.pmed.1003093 (PMC7108713; doi:10.1371/journal.pmed.1003093)
Supplement: S6 Table — (DOCX) [file pmed.1003093.s006.docx]

S6 Table Replication of Table 3 using an ordered logit model

|  | (1) | (2) | (3) | (4) |
| --- | --- | --- | --- | --- |
|  | OLogit | OLogit | OLogit | OLogit |
| SES at T0 | 0.60^***^ | 0.59^***^ | 0.59^***^ | 0.58^***^ |
|  | [0.50,0.69] | [0.49,0.69] | [0.49,0.69] | [0.48,0.69] |
| T1 | 0.54^***^ | 0.56^***^ | 0.56^***^ | 0.55^***^ |
|  | [0.29,0.80] | [0.30,0.82] | [0.29,0.82] | [0.28,0.82] |
| SES x T1 | -0.48^***^ | -0.49^***^ | -0.49^***^ | -0.49^***^ |
|  | [-0.59,-0.37] | [-0.60,-0.38] | [-0.60,-0.38] | [-0.60,-0.38] |
| Sociodemographics | No | Yes | Yes | Yes |
| Migration experience | No | No | Yes | Yes |
| Experience in Germany | No | No | No | Yes |
| *Number of observations* | 4302 | 4162 | 4162 | 4128 |
| pseudo *R*^2^ | 0.03 | 0.04 | 0.04 | 0.04 |
| Notes: Dependent variable for all regression: health satisfaction. Results based on an ordered logit model. Covariates included in all regressions: sex, age, age². Sociodemographics: marital status, income at T0, educational attainment at T0, number of children, Syrian birth region dummies. Migration experience: neg. migration experience, duration of migration. Experience in Germany: employment status at T1, feeling of welcome, year of arrival. 95% CIs based on heteroskedastic robust standard errors clustered on the individuum in brackets. * p < 0.1, ** p < 0.05, *** p < 0.01. | | | | |
