# Supplementary material for: Pre-migration socioeconomic status and post-migration health satisfaction among Syrian refugees in Germany: A cross-sectional analysis
Source: PLoS Med. 2020 Mar 31;17(3):e1003093. doi: 10.1371/journal.pmed.1003093 (PMC7108713; doi:10.1371/journal.pmed.1003093)
Supplement: S7 Table — (DOCX) [file pmed.1003093.s007.docx]

S7 Table Regression underlying Fig 3 & Fig 4

|  | (1) | (2) |
| --- | --- | --- |
|  | Men | Women |
| SES in T0 | 0.66*** | 0.53*** |
|  | [0.51,0.80] | [0.35,0.71] |
| T1 | 0.84*** | -0.06 |
|  | [0.43,1.25] | [-0.64,0.52] |
| SES x T1 | -0.54*** | -0.39*** |
|  | [-0.70,-0.38] | [-0.63,-0.15] |
| Age | -0.03 | -0.03 |
|  | [-0.09,0.03] | [-0.11,0.05] |
| Age² | -0.00 | -0.00 |
|  | [-0.00,0.00] | [-0.00,0.00] |
| Sociodemographics | Yes | Yes |
| Migration experience | Yes | Yes |
| Experience in Germany | Yes | Yes |
| *N* | 2656 | 1472 |
| adj. *R*^2^ | 0.10 | 0.14 |
| Notes: Dependent variable for all regression: health satisfaction. Results based on OLS. Covariates included in all regressions: sex, age, age². Sociodemographics: marital status, income at T0, educational attainment at T0, number of children, Syrian birth region dummies. Migration experience: neg. migration experience, duration of migration. Experience in Germany: employment status at T1, feeling of welcome, year of arrival. 95% CIs based on heteroskedastic robust standard errors clustered on the individuum in brackets. * p < 0.1, ** p < 0.05, *** p < 0.01. | | |
