# Supplementary material for: Pre-migration socioeconomic status and post-migration health satisfaction among Syrian refugees in Germany: A cross-sectional analysis
Source: PLoS Med. 2020 Mar 31;17(3):e1003093. doi: 10.1371/journal.pmed.1003093 (PMC7108713; doi:10.1371/journal.pmed.1003093)
Supplement: S8 Table — (DOCX) [file pmed.1003093.s008.docx]

S8 Table Life satisfaction similar to Table 3

|  | (1) | (2) | (3) | (4) | (5) |
| --- | --- | --- | --- | --- | --- |
|  | OLS | OLS | OLS | OLS | FE |
| SES at T0 | 1.15*** | 1.15*** | 1.15*** | 1.14*** |  |
|  | [1.04,1.26] | [1.04,1.27] | [1.04,1.27] | [1.02,1.26] |  |
| T1 | 2.05*** | 2.05*** | 2.05*** | 2.05*** | 2.05*** |
|  | [1.68,2.43] | [1.67,2.43] | [1.67,2.43] | [1.67,2.44] | [1.67,2.42] |
| SES x T1 | -1.09*** | -1.09*** | -1.09*** | -1.10*** | -1.09*** |
|  | [-1.24,-0.94] | [-1.24,-0.94] | [-1.24,-0.94] | [-1.25,-0.95] | [-1.23, -0.94] |
| Male | -0.40*** | -0.34*** | -0.34*** | -0.36*** |  |
|  | [-0.54,-0.26] | [-0.51,-0.18] | [-0.50,-0.17] | [-0.53,-0.20] |  |
| Age | -0.04** | -0.08*** | -0.08*** | -0.09*** |  |
|  | [-0.08,-0.00] | [-0.13,-0.03] | [-0.13,-0.03] | [-0.14,-0.05] |  |
| Age² | 0.00 | 0.00** | 0.00** | 0.00*** |  |
|  | [-0.00,0.00] | [0.00,0.00] | [0.00,0.00] | [0.00,0.00] |  |
| Sociodemographics | No | Yes | Yes | Yes | No |
| Migration experience | No | No | Yes | Yes | No |
| Experience in Germany | No | No | No | Yes | No |
| Number of observations | 4289 | 4149 | 4149 | 4119 | 4291 |
| adj. R2 | 0.11 | 0.12 | 0.12 | 0.13 | 0.11 |
| Notes: Dependent variable for all regression: life satisfaction. Results in column 1-4 based on OLS. Socio-demographics: marital status, income at T0, educational attainment at T0, number of children, Syrian birth region dummies. Migration experience: neg. migration experience, duration of migration. Experience in Germany: employment status at T1, feeling of welcome, year of arrival. Column 5 based on within-estimator accounting for individual fixed-effects. 95% CIs based on heteroskedastic robust standard errors clustered on the individuum in brackets. * p < 0.1, ** p < 0.05, *** p < 0.01. | | | | | |
