# Supplementary material for: Pre-migration socioeconomic status and post-migration health satisfaction among Syrian refugees in Germany: A cross-sectional analysis
Source: PLoS Med. 2020 Mar 31;17(3):e1003093. doi: 10.1371/journal.pmed.1003093 (PMC7108713; doi:10.1371/journal.pmed.1003093)
Supplement: S9 Table — (DOCX) [file pmed.1003093.s009.docx]

S9 Table. Tests for the importance of ceiling effects.

|  | (1) | (2) | (3) | (4) |
| --- | --- | --- | --- | --- |
|  | OLS | OLS | Tobit | Tobit |
|  | Full sample | No ceiling sample |  | With Ln of outcome |
| main |  |  |  |  |
| SES in T0 | 0.64*** | 0.53*** | 1.25*** | 0.17*** |
|  | [0.53,0.75] | [0.39,0.67] | [1.04,1.45] | [0.14,0.20] |
| T1 | 0.50*** | 1.30*** | 1.13*** | 0.16*** |
|  | [0.17,0.82] | [0.90,1.69] | [0.59,1.66] | [0.08,0.24] |
| SES x T1 | -0.48*** | -0.36*** | -0.99*** | -0.14*** |
|  | [-0.61,-0.35] | [-0.53,-0.19] | [-1.21,-0.76] | [-0.18,-0.11] |
| N | 4302 | 3209 | 4302 | 4218 |
| adj./pseudo R2 | 0.09 | 0.08 | 0.03 | 0.06 |
| Notes: Dependent variable in model 1-3 is health satisfaction. Model 4 uses the log transformation. All regressions use sex and age as a control. 95% CIs based on heteroskedastic robust standard errors clustered on the individuum in brackets. * p < 0.1, ** p < 0.05, *** p < 0.01. | | | | |
