# Supplementary material for: Pre-migration socioeconomic status and post-migration health satisfaction among Syrian refugees in Germany: A cross-sectional analysis
Source: PLoS Med. 2020 Mar 31;17(3):e1003093. doi: 10.1371/journal.pmed.1003093 (PMC7108713; doi:10.1371/journal.pmed.1003093)
Supplement: S1 Fig — (DOCX) [file pmed.1003093.s010.docx]

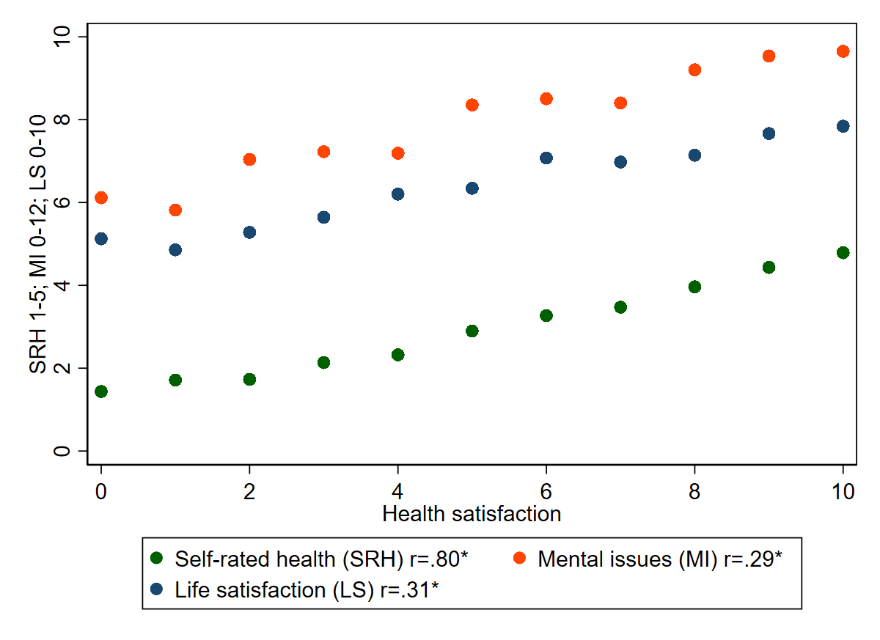


S1 Fig. Relationship between health satisfaction and other indicators.

R reflects the spearman correlation coefficient with health satisfaction at T1. The MH scale was inverted. Y-title shows total range of the variable. * indicates a p-value<0.05.
