# Supplementary material for: Pre-migration socioeconomic status and post-migration health satisfaction among Syrian refugees in Germany: A cross-sectional analysis
Source: PLoS Med. 2020 Mar 31;17(3):e1003093. doi: 10.1371/journal.pmed.1003093 (PMC7108713; doi:10.1371/journal.pmed.1003093)
Supplement: S2 Fig — (DOCX) [file pmed.1003093.s011.docx]

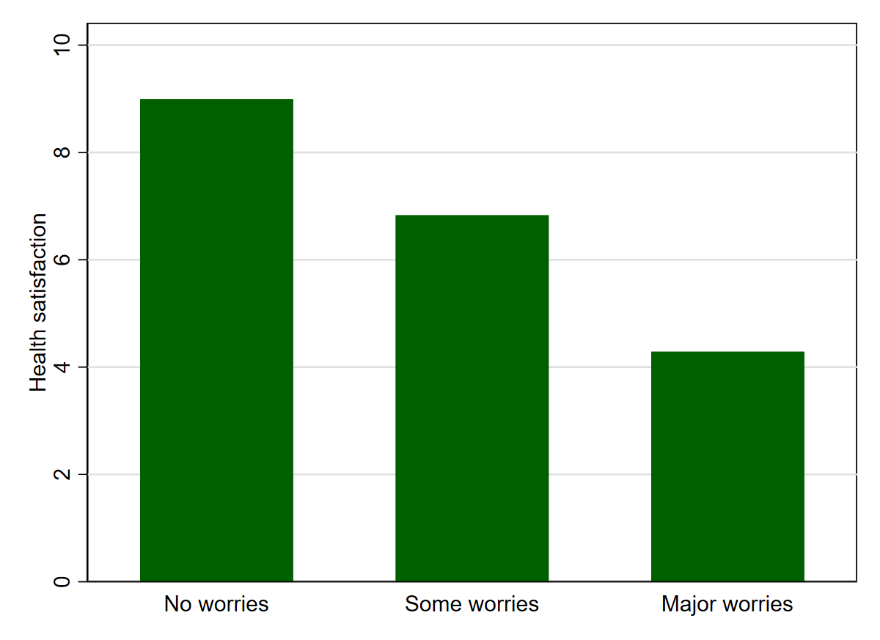


S2 Fig. Relationship between health worries and health satisfaction

Bar chart plotting *worries with health* against Health satisfaction at T1

| . |
| --- |
