# Supplementary material for: Pre-migration socioeconomic status and post-migration health satisfaction among Syrian refugees in Germany: A cross-sectional analysis
Source: PLoS Med. 2020 Mar 31;17(3):e1003093. doi: 10.1371/journal.pmed.1003093 (PMC7108713; doi:10.1371/journal.pmed.1003093)
Supplement: S2 Data — (DOCX) [file pmed.1003093.s014.docx]

| **Variable in Paper (mostly Table 1)** | **Variable Name in Do-File** | | **DIW Codebook PDF*** | |
| --- | --- | --- | --- | --- |
|  | Original | Recoded | Question | Page |
| Male | bgpr_l_0101 | male | B001/PA 3 | 103 |
| Age | bgpr_l_0103 | age | B001/PA2 | 103 |
| Syrian region | bgpr_l_0319 | area_syria | B003/3t | 117 |
| Marital status | bgpr390 | marital_stat | b050/390 | 217 |
| Number of children | bgpr_l_402 | number_children | b050/402 | 219 |
| Educational attainment | bgpr_l_227 | education | b033/227 | 176 |
| Income | bgpr_l_15201 | income | b028/152 | 157 |
| Needed for Income | bgpr_l_151 | income | b028/151 | 157 |
| Year of arrival | bgpr_l_3401 | year_immigration_ger | B006/34 | 128 |
| Needed for Duration of migration | bgpr_l_0501 | year_immigration_first | B006/5 | 121 |
| Health satisfaction at T0 | bgpr_l_159 | hs0 | b028/159 | 159 |
| Health satisfaction at T1 | bgpr298 | hs1 | b041/298 | 187 |
| Life satisfaction at T0 | bgpr_l_160 | ls0 | b028/160 | 160 |
| Life satisfaction at T1 | bgpr457 | ls1 | b050a/457 | 224 |
| Subjective socioeconomic status | bgpr_l_157 | ses | b028/ 157 | 159 |
| Needed for Unemployed | bgpr161 | cur_employment_stat | b029/161 | 160 |
| Number of negative experiences (sum) | | |  |  |
| Financial fraud or financial exploitation | bgpr_l_3301 | bgpr_l_3301 | B006/33 | 127 |
| Sexual harassment | bgpr_l_3302 | bgpr_l_3302 | B006/33 | 127 |
| Physical attacks | bgpr_l_3303 | bgpr_l_3303 | B006/33 | 127 |
| Shipwreck | bgpr_l_3304 | bgpr_l_3304 | B006/33 | 127 |
| Robbery | bgpr_l_3305 | bgpr_l_3305 | B006/33 | 127 |
| Blackmail | bgpr_l_3306 | bgpr_l_3306 | B006/33 | 127 |
| Imprisonment | bgpr_l_3307 | bgpr_l_3307 | B006/33 | 127 |
| Feeling welcome | bgpr326 | welcome_arrival | b042/326 | 193 |
| Worries about health | bgpr355 | health_worry | B044/354 | 200 |
| Self-rated health | bgpr299 | health_status | b041/299 | 187 |
| Needed for Mental health | bgpr312 | bgpr312 | b041/312 | 190 |
| Needed for Mental health | bgpr313 | bgpr313 | b041/313 | 190 |
| Needed for Mental health | bgpr314 | bgpr314 | b041/314 | 190 |
| Needed for Mental health | bgpr315 | bgpr315 | b041/315 | 190 |
| *Codebook available from: <https://www.diw.de/documents/publikationen/73/diw_01.c.570916.de/diw_ssp0362.pdf> | | | | |
