# Supplementary material for: Pre-migration socioeconomic status and post-migration health satisfaction among Syrian refugees in Germany: A cross-sectional analysis
Source: PLoS Med. 2020 Mar 31;17(3):e1003093. doi: 10.1371/journal.pmed.1003093 (PMC7108713; doi:10.1371/journal.pmed.1003093)
Supplement: Attachment — Submitted filename: Review PLoS Medicine.docx [file pmed.1003093.s015.docx]

Review PLoS Medicine: Pre-migration socioeconomic status and post-migration health: evidence from Syrian refugees in Germany.

This is a paper that is of high interest for people in the field and policy-makers. It is well-written and well-conducted. I have some comments that could possibly ameliorate this paper.

**Major Comments:**

- Please add some references to the first paragraph.

- p. 2, line 51: please explain why did you then decide to use individual SES and not at the household level?

- Please explain the S&W concerning the use of self-reported SES, e.g. over-or underestimation,…

- p. 6: you include education as a covariate but isn’t it a measure of SES?

- please explain why you didn’t stratify all the analyses by gender as both self-reported SES and self-reported health may differ by gender.

- Please discuss the age limits of the study.

- p. 9: please explain the category of education ‘certificate different’.

- p. 9: please discuss gender differences in the descriptives as well.

- The tables should be readable and easily interpretable on itself. Please explain in the titles and headings what numbers are shown.

- The figures are not readable.

- As the dependent variables are self-reported health variables, I personally think that these observed differences are not so much related to access to health care but more to experiences along the way. Please discuss this issue in the discussion paragraph.

- p. 17, line 352: as this is a cross-sectional study, please be careful when using words as ‘influences’.

**Minor Comments:**

- p. 2, lines 28-30: please check the construction of this sentence.

- p. 2 line 34: please first write SES in full spell before using the abbreviation.

- p. 2, line 45: lead to.

- p. 3, line 58: pathways,

- p. 3, line 76: SES instead of SESs.

- p. 4, lines 96 and 100: data were instead of data was.

- p. 5, line 129: please check the construction of this sentence.

- p. 9, line 201: happened.

- p. 17, line 341: our data suggest.

- p. 19, line 390: specific points in time.
