# Supplementary material for: Pre-migration socioeconomic status and post-migration health satisfaction among Syrian refugees in Germany: A cross-sectional analysis
Source: PLoS Med. 2020 Mar 31;17(3):e1003093. doi: 10.1371/journal.pmed.1003093 (PMC7108713; doi:10.1371/journal.pmed.1003093)
Supplement: Attachment — Submitted filename: Pointbypointreply.docx [file pmed.1003093.s016.docx]

**Response to the editor and reviewers**

We would like to thank the editor and the three reviewers for their insightful comments that helped to clarify and improve the manuscript. Please find a point-by-point reply in this document below.

|  | **Comments from the editor:** |
| --- | --- |
| 1 | Please revise your title according to PLOS Medicine's style. Your title must be nondeclarative and not a question. It should begin with main concept if possible. "Effect of" should be used only if causality can be inferred, i.e., for an RCT. Please place the study design ("A randomized controlled trial," "A retrospective study," "A modelling study," etc.) in the subtitle (ie, after a colon). |
|  | *Response: We changed the title to “Pre-migration socioeconomic status and post-migration health satisfaction among Syrian refugees in Germany: a retrospective analysis”.* |
| 2 | Please structure your abstract using the PLOS Medicine headings (Background, Methods and Findings, Conclusions).  Abstract Background: Provide the context of why the study is important. The final sentence should clearly state the study question.  Abstract Methods and Findings:  * Please ensure that all numbers presented in the abstract are present and identical to numbers presented in the main manuscript text.  * Please include the study design, population and setting, number of participants, years during which the study took place, length of follow up, and main outcome measures.  * Please quantify the main results (with 95% CIs and p values).  * Please include the important dependent variables that are adjusted for in the analyses.  * Please include the actual amounts and/or absolute risk(s) of relevant outcomes (including NNT or NNH where appropriate), not just relative risks or correlation coefficients. (example for absolute risks: PMID: 28399126).  * Please include a summary of adverse events if these were assessed in the study.  * In the last sentence of the Abstract Methods and Findings section, please describe the main limitation(s) of the study's methodology. |
|  | *Response: We changed the abstract accordingly.* |
| 3 | Data – the URL you provide does not seem to take one to a repository. All data needs to be made available – and for referees to also assess. Please ensure this is available on resubmission. Noting an author cannot be a point of contact. Also remove the reference to data and on request from line 102. |
|  | *Response:*  *We would like to clarify that for this study we use data that we did not collect ourselves, but which was collected by the DIW. The SOEP is freely available for scientific use but requires the filling-in of an application form. The procedure is explained in detail here:* [*https://www.diw.de/documents/dokumentenarchiv/17/diw_01.c.44985.de/soep_cnef_access_procedures_2014.pdf*](https://www.diw.de/documents/dokumentenarchiv/17/diw_01.c.44985.de/soep_cnef_access_procedures_2014.pdf)  *We are however contractually not allowed to upload the data ourselves. We got in touch with the DIW, the owner of data, and were assured that every researcher who wants to replicate or check our analysis will be given access to the data upon request. We understand that this is not exactly the same level of accessibility as uploading the raw data into a repository, but we hope this is a feasible solution for the publication process. The statistical code starts from the raw data as provided by the DIW and hence can be easily replicated.* |
| 4 | At this stage, we ask that you include a short, non-technical Author  Summary of your research to make findings accessible to a wide  audience that includes both scientists and non-scientists. The  Author Summary should immediately follow the Abstract in your  revised manuscript. This text is subject to editorial change and  should be distinct from the scientific abstract. Please see our  author guidelines for more information:  <https://journals.plos.org/plosmedicine/s/revising-your-manuscript#lo> |
|  | *Response: We have included the Summary in the main document.* |
| 5 | c- author-summary Please ensure all questionnaires are provided as  Supp files (and translated into English, as needed). |
|  | *Response: The DIW provides an extensive codebook with all questions in German and English. We thought it would be most helpful to guide interested researchers by providing a document that links our survey questions to this codebook. We attached this to our submission* |
| 6 | > Line 249 – where (not were) |
|  | *Response: This has been changed accordingly.* |
| 7 | All of your Figs appear to have converted oddly and appear very small. Please do visit our guidelines on figures format and size. |
|  | *Response: We have adjusted the figure sizes and now provide tif-files in sufficient pixels along with the material* |
| 8 | Please ensure that the study is reported according to the [STROBE] guideline, and include the completed [STROBE or other] checklist as Supporting Information. When completing the checklist, please use section and paragraph numbers, rather than page numbers. Please add the following statement, or similar, to the Methods: "This study is reported as per the Strengthening the Reporting of Observational Studies in Epidemiology (STROBE) guideline (S1 Checklist)."  Please report your study according to the relevant guideline, which  can be found here: <http://www.equator-network.org/> |
|  | *Response: We filled out the STROBE form and added it as supplementary material.* |

|  | **Comments from the reviewers:** |
| --- | --- |
|  | **Reviewer #1:** "Pre-migration socioeconomic status and post-migration health: evidence from Syrian refugees in Germany" analyzes survey data of 2000-plus Syrian refugees that had entered Germany on or after 2013, using two main standard multivariate regression models - the first a cross-sectional analysis for five health measures for their current status in Germany (T1), and the second a longitudinal analysis also involving their condition when in Syria (T0).  > The authors modify both main regression models with varying combinations of covariates (results summarized in Tables 2 & 3) such as sex, age, sociodemographics, migration experience & experience in Germany, which generally produce similar (robust) outcomes, with the major exception of sex (covered in the section on Heterogeneity). The key conclusion is that the main dependant variable of after-migration "health satisfaction" is strongly attentuated (significantly less strongly correlated) with reference to pre-migration SES, in contrast to pre-migration "health satisfaction" (expectedly?) being highly correlated with SES, as visually displayed in Figures 1 & 2. |
| 9 | ) A first observation would be that the conclusions from the current analysis might more properly be claimed on "post-migration health satisfaction" rather than "post-migration health" (as in the manuscript title), as the relevant longitudinal analysis was performed on "health satisfaction" and not "(self-reported) health", notwithstanding the relatively high (~0.8) correlation between the two indicators as shown in Table A1. |
|  | *Response: We agree with the reviewer and have changed the title accordingly.* |
| 10 | Moreover, the correlation with "mental distress (mental health?)" is weak. The distinction between "health satisfaction" and (actual) "self-reported health" would appear to be important, because there likely remain interactions between expressed satisfaction and underlying health that are difficult to quantify (e.g. one's health is so-so, but he thinks it reasonable given his current state as a refugee, and thus expresses satisfaction). There might be latitude to visualize the typical relationship between "health satisfaction" and "self-reported health" in a manner similar to Figures 1 & 2. |
|  | Response:  We have now added the above figure in the Appendix to show the clear relationship between the conditional means of the other variables and health satisfaction. The trends are clearly similar, with a stronger relationship for SRH. |
| 11 | 2) Following on, a possible approach for analyzing "self-reported health" longitudinally, might be to substitute the missing T0 "self-reported health" data, with values predicted using T0 "health satisfaction" and the relationship between T1 "health satisfaction" and T1 "self-reported health", assuming this relationship stays constant. |
|  | *Response: This is an interesting idea. Nevertheless, we would rather stick with the health satisfaction variable because we think that the relationship between health satisfaction and self-rated health could have been different before migration because, as the reviewer has pointed out in the statement before, it may reflect their status as a normal citizen (and not the status as a refugee). We however tested the suggestions predicting self-rated health at T0 with a regression of health satisfaction and clearly exogenous variables (age, gender). The results remain very similar to the ones for health satisfaction given their high correlation. See below and in contrast to Figure 1 in the paper.*   |
| 12 | 3) Is there a particular justification for adding the "sociodemographics", then "migration experience", then "experience in Germany" covariates in that order, for both the cross-sectional and longitudinal models? |
|  | *Response: The order of the inclusion was intended to reflect the temporal order of the statuses or events: situation before migration (T0), during migration (migration experience) and post migration (experience in Germany). Nevertheless, since the main association does not change substantially, the order of the included covariates does not affect the results. We added a clarifying sentence to the text: “*From Panel A to D we add variables that could mitigate the relationship of interest in a chronological order starting with characteristics set before their migration experience, followed by assessments of the migration period itself and variables capturing their experience in Germany.” |
| 13 | 4) The authors note possible ceiling effects of a finite scale in their discussion of limitations. It is known that individuals may subjectively treat the scales in different ways (e.g. some might use only the top few values, while others might use the full scale). Might any of the (possibly unused) survey responses be used to estimate such tendencies? The authors may wish to discuss this possible effect further. |
|  | *Response: We present two approaches to address the importance of these ceiling effects and provide their results in the Appendix. The individual specific tendencies to use such scales is a general survey problem that should however play a minor role in our main analysis as we assess differences between two time periods in a one-dimensional measure. These within-subject differences in response from T0 and T1 should be less affected by individual response behavior that might be correlated with other outcomes. We present different tests in the paper:* 1. *We provide the estimates for a regression only using people with baseline health satisfaction between 1-9. So, their outcome can move in either direction. The results remain unchanged.* 2. *We went through the related literature followed the approach by Huang et al. (2008) and estimated two tobit models that replicated the dynamic from the main results.* *Huang, I. C., Frangakis, C., Atkinson, M. J., Willke, R. J., Leite, W. L., Vogel, W. B., & Wu, A. W. (2008). Addressing ceiling effects in health status measures: A comparison of techniques applied to measures for people with HIV disease. Health Services Research, 43, 327–339.* *We tried to follow the idea suggested by the reviewer but struggled to find peer-reviewed literature on the exact method. We however would like to present some findings here. We looked at a 6-item scale on reciprocity which is uncorrelated with our outcome (spearman, average reciprocity score and health satisfaction in t =1, r= 0.01, P=0.51). Counting the number of endpoint responses in these 6 items (either lowest or highest point on the scale) predicted higher scores for health satisfaction but did not change our estimates.* \| (1) \| (2); \| OLS Full sample \| OLS Controlled for use of scale endpoints; SES at T0 \| 0.6432*** \| 0.6459***; \| (0.06) \| (0.06); T1 \| 0.4960*** \| 0.4979***; \| (0.17) \| (0.17); SES x T1 \| -0.4763*** \| -0.4769***; \| (0.07) \| (0.07); 0 endpoints \| \| .; \| \| .; 1 endpoint \| \| 0.6574**; \| \| (0.30); 2 endpoints \| \| 0.6569***; \| \| (0.25); 3 endpoints \| \| 0.8825***; \| \| (0.24); 4 endpoints \| \| 0.8181***; \| \| (0.24); 5 endpoints \| \| 0.9528***; \| \| (0.22); 6 endpoints \| \| 1.1312***; \| \| (0.22); *N* \| 4302 \| 4302; adj. *R*^2^ \| 0.09 \| 0.10; Notes: Endpoint measure refers to a count variable for number of used endpoints of a 7-point scale regarding reciprocity. \| \| |
| 14 | 5) In Equation 1, what does the Beta_1a term refer to? |
|  | *Response: We apologise for the omission. Beta 1 in regression equation 1 indicated the intercept. We saw that we did not do this in regression equation 2. To harmonize this, we have now removed beta_1 from the first equation and clarify this similarly in the text.* |
| 15 | 6) There are some minor grammatical/phrasing issues, e.g. "...the fact that migrant often live" (Line 14), "During migration passage... but also their legal status, housing conditions, economic prospects and the contexts of reception." (Line 23-28); also there is an occasional inconsistency with citation practices, for example "Spallek and colleagues (2011)..." in Line 20, with appears without the standard bracketed reference to [8] and/or [9]. |
|  | *Response: We apologize for these errors – the manuscript has now been thoroughly proofread by a native speaker.* |
|  |  |
|  | **Reviewer #2:** Review PLoS Medicine: Pre-migration socioeconomic status and post-migration health: evidence from Syrian refugees in Germany.  This is a paper that is of high interest for people in the field and policy-makers. It is well-written and well-conducted. I have some comments that could possibly ameliorate this paper. |
| 16 | Major Comments: Please add some references to the first paragraph |
|  | *Response: We have now added more references to this paragraph.* |
| 17 | p. 2, line 51: please explain why did you then decide to use individual SES and not at the household level? |
|  | *Response: We agree that an objective SES measure would have been preferable. Unfortunately, there is no household SES measure in this dataset.* |
| 18 | Please explain the S&W concerning the use of self-reported SES, e.g. over-or underestimation,… |
|  | *Response: Here we follow the seminal work by Nancy Adler (2000) showing that there is predicitive power in subjective data beyond the acutal hard indicators. Others have provided opposite results (e.g. Macleod et al.) Even when subjective data is more predictive, one might be worried about bias. According to Cundiff et al., subjective SES and subjective reports of health could show stronger correlation due to common method variance or reporting bias.*  *However, we did not find clear evidence for this to be a major issue with studies suggesting that " subjective social status reflects the cognitive averaging of standard markers of socioeconomic situation and is free of psychological biases.” (Singh_Manoux et al.)*  *This point has now been emphasized in the methods section where we present the measures.*  *Adler NE, Epel E, Casterllazzo G, Ickovics J. Relationship of subjective and objective social status with psychological and physical health in healthy white women. Heal Psychol. 2000;19: 586–592.*  *Cundiff, J. M., & Matthews, K. A. (2017). Is subjective social status a unique correlate of physical health? A meta-analysis. Health Psychology, 36(12), 1109–1125. https://doi.org/10.1037/hea0000534*  *Macleod, J., Davey, G., Metcalfe, C., & Hart, C. (2005). Is subjective social status a more important determinant of health than objective social status ? Evidence from a prospective observational study of Scottish men. 61, 1916–1929.*  *Singh-Manoux, A., Adler, N. E., & Marmot, M. G. (2003). Subjective social status : its determinants and its association with measures of ill-health in the Whitehall II study. 56, 1321–1333.* |
| 19 | p. 6: you include education as a covariate but isn't it a measure of SES? |
|  | *Response: We included education as well as income as objective measures for SES because we wanted to analyze whether the association of subjective SES with health satisfaction is independent of objective SES indicators as suggested by some other studies. Adding such objective variables reduces the effect but does not fully explain it. These results provide additional information to the discussion above and we think it is worth showing them, even though we agree with the reviewer that adding education could be considered as “over controlling” or a “bad control”.* |
| 20 | please explain why you didn't stratify all the analyses by gender as both self-reported SES and self-reported health may differ by gender. |
|  | *Response: We decided to keep the full sample for the main analysis to have maximum statistical power. Stratification by gender was planned as an additional analysis to check the consistency of the results. When looking at the gender stratified results, one could argue that the whole analysis should be done separately for men and women, but we wanted to present the results in the order that we originally planned. We also think that by presenting the gender specific results in an extra figure und table the differences become clearer.* |
| 21 | - Please discuss the age limits of the study. |
|  | *Response:* The study covers a broad age range (18-75 years). However, only a small proportion (about 10%) is older than 50 years. (Unaccompanied) minors – a group that is perceived as very vulnerable - are not included in this study. We have now added this as a limitation in the discussion section. |
| 22 | p. 9: please explain the category of education 'certificate different'. |
|  | *Response: We have now changed the description to “other certificate”. The original category in the survey was named “certificate from different school” as some sort of residual category. There is unfortunately no additional information.* |
| 23 | p. 9: please discuss gender differences in the descriptives as well. |
|  | *Response: We have now added two columns for women and men to the descriptive table and added a brief discussion about the differences.* |
| 24 | The tables should be readable and easily interpretable on itself. Please explain in the titles and headings what numbers are shown. |
|  | *Response: We have changed the formats of the tables and figures and extended the titles to improve the readability.* |
| 25 | The figures are not readable |
|  | *Response: The figures have now been replaced in a better format.* |
| 26 | - As the dependent variables are self-reported health variables, I personally think that these observed differences are not so much related to access to health care but more to experiences along the way. Please discuss this issue in the discussion paragraph. |
|  | *Response: We agree that access to healthcare does not fully explain the observed differences. In our view this has more to do with psychosocial factors such as status inconsistencies (see discussion 4^th^ paragraph line 445 onwards. Experiences during migration are important but we controlled for them in the analysis.* |
| 27 | p. 17, line 352: as this is a cross-sectional study, please be careful when using words as 'influences'. |
|  | *Response: We have changed this into “…*the leveling of the association between pre-migration SES and health satisfaction” |
| 28 | Minor Comments:  - p. 2, lines 28-30: please check the construction of this sentence.  - p. 2 line 34: please first write SES in full spell before using the abbreviation.  - p. 2, line 45: lead to.  - p. 3, line 58: pathways,  - p. 3, line 76: SES instead of SESs.  - p. 4, lines 96 and 100: data were instead of data was.  - p. 5, line 129: please check the construction of this sentence.  - p. 9, line 201: happened.  - p. 17, line 341: our data suggest.  - p. 19, line 390: specific points in time. |
|  | *Response: We apologize for these errors – the manuscript has now been thoroughly proofread by a native speaker.* |
|  |  |
|  | **Reviewer #3:** Overall this is a useful study using a large dataset, and provides novel findings on the complex relationship between SES and mental health. I have listed minor points below, the majority of which relate to a lack of polished language/descriptions and multiple typos. Once these are addressed, I think this study would be well worthy of publication. |
| 29 | Abstract  I would suggest avoiding using the word 'influx' as this has become  a politicized word Change 'on the top' to 'at the top'  Please name the study design  Line 8 amend 'their' to 'migrant'  I think it is misleading to refer to a longitudinal analysis when data was only collected at one time point? Could it be referred to as a retrospective analysis? |
|  | *Response: We agree that it is more appropriate to describe this as a retrospective analysis. We have replaced ‘influx’ with ‘this large increase’ and changed ‘on the top’ to ‘at the top’.* |
| 30 | Introduction  P1 line 4 - can the authors define what 'top country' refers to? Country with the most migrants?  P1 line 14 - change migrant to migrants  P2 line 34 -please define the acronym in the first use  P2 line 45 - leads instead of lead  P3 line 57 - 'the' chronically ill  P4 line 80 - please state the study design  P4 line 81 - the question 'of'  P5 line 87 - 'a' scientific  > > |
|  | *Response:’ Top countries’ refer to the absolute number of international migrants in the country. We clarified this in the text. We apologize for the language errors and have changed the words accordingly.* |
| 31 | P4 line 88-91 - the hypotheses could be clearer. What is meant by safeguard? As we don't know the study design or what kind of data will be used, it's hard to know what this means. The first hypothesis also seems to include two hypothesis within it. |
|  | Response: The study is set up to provide empirical support to one of the two competing hypotheses. From the literature and the discussed theories, we cannot predict if SES differences become more pronounced or are attuned. We try to emphasize this in the introduction chapter. An example of a similar approach can be found in:  Falk, A., & Hermle, J. (2018). Relationship of gender differences in preferences to economic development and gender equality. Science, 362.  In response to the reviewers comment we clarified this part and briefly elaborate on the not so easily explained method used. We hope this reduces confusion for the reader. |
| 32 | Methods  P4 line 93 - again, the study design needs to be explicit from the  start, before describing where the data comes from |
|  | *Response: We added a sentence at the start of the methods section.* |
| 33 | P4 line 99 - change 'their' to migrant'  P5 line 114 - as 'an' indicator  P5 line 120 - 'used' not 'use'  P5 line 124 - 'was' measured  P6 line 140 - sentence doesn't make sense  P6 line 155 - for a 'full' description  P7 line 157 - change 'their' to 'migrant' |
|  | *Response: These have been changed accordingly.* |
| 34 | P4 line 93 - international readers, like me, will not be familiar with the SOEP. Could the authors add a sentence to describe what it is? |
|  | *Response: We apologize for the omission and now present a few more introductory sentences about the SOEP, its background and the link to the data used in the present study. We have also added a number of references that provide an extensive overview for readers that want to know more.* |
| 35 | P4 line 95 - why predominantly in these dates? Can the authors be specific about which dates are included? |
|  | *Response: As is now mentioned in the text, the data were collected in response to the publicly discussed “refugee crisis” in 2015, where more than 1,000,000 refugees came to Germany. The data collection is elaborated on in Kühne et al. and random sampling was done in the following year. However, refugees from Syrian, at the core of this study, virtually all arrived during this time period. As we are confident that refugees living in Germany for a longer time had a very different experience or memory of their lives before the migration, we excluded 20 people. This way we ensured that the retrospective assessment referred to similar time frames.*  Kühne, S., Jacobsen, J., & Kroh, M. (2019). Sampling in Times of High Immigration: The Survey Process of the IAB-BAMF-SOEP Survey of Refugees. *Survey Methods: Insights from the Field (SMIF)*, 1–9. |
| 36 | P4 line 103 the link is helpful but I think more detail should be in this section. How were the migrants identified by the system? How were they first contacted? How was an interpreter arranged? As it stands, it is quite unclear how these data was generated. It is also unclear what the overall pupose of data collection was, and exactly what was included. |
|  | *Response: We have added some details about the methodology but would like to again refer to Kühne et al. for an excellent summary of the survey methods.*    Kühne, S., Jacobsen, J., & Kroh, M. (2019). Sampling in Times of High Immigration: The Survey Process of the IAB-BAMF-SOEP Survey of Refugees. *Survey Methods: Insights from the Field (SMIF)*, 1–9. |
| 37 | P5 line 126 - it might be worth citing studies that show the PHQs  validity in migrant populations too?  <https://www.ncbi.nlm.nih.gov/pubmed/30156742> |
|  | *Response: Thank you for the hint, we included this information in this section.* |
| 38 | Results: P9 table 1 - the table is a little unclear, what does Income N/A mean and why is the % so high? Given that income is a core part of SES it seems an important variable to be missing almost half of? |
|  | *Response: We elaborate on the unavailability of this metric in the in the methods section. From the gender split analysis (Table 1), one can see that predominantly women report no income, who might just not be part of the labor market.* |
| 39 | P10 line 210 - sentence does not make sense  P12 line 244 - rephrase 'lower levels of health' Its unclear what  this means  P12 line 248 - rephrase pre-migration 'area' |
|  | *Response: We have changed these sentences.* |
| 40 | Discussion  P17 line 335 - people who have been trafficked instead of traffic  persons  P17 line 342-43 - rephrase as it does not make sense  P19 line 389 - 'a' point in time  P19 line 404 - 'Syrians' or 'Syrian migrants' not 'Syrian' |
|  | *Response: We have changed these sentences.* |
| 41 | P18 line 381 - the two time points seems misleading, isn't it retrospective? The wording here (and in thr abstract) implies to points of administration of measures? It's also unclear how missing data was handled. |
|  | *Response: Following the recommendation by reviewer #1 and the editor, we renamed the study to avoid confusion and clarified the respective passages in the abstract and the text. We have added a sentence in the analysis section that explains the handling of missing data.* |
