# Supplementary material for: Pre-migration socioeconomic status and post-migration health satisfaction among Syrian refugees in Germany: A cross-sectional analysis
Source: PLoS Med. 2020 Mar 31;17(3):e1003093. doi: 10.1371/journal.pmed.1003093 (PMC7108713; doi:10.1371/journal.pmed.1003093)
Supplement: Attachment — Submitted filename: Pointbypointreply.docx [file pmed.1003093.s017.docx]

Point by point Reply 2^nd^ Round

------------------------------------------------------------

Requests from Editors:

| 1 | Data- the link [www.diw.de](http://www.diw.de) appears to be a general site. It is not clear how one can access data from this. Please provide a direct link to the data from your study. Also data on request (one presumes from the authors) does not comply with our open data policy where authors cannot be points of contact. Please provide the contact details of say a data committee where the method/ code can be accessed from. |
| --- | --- |
|  | *Response: Based on a recent study published in PLOS Med.* <https://journals.plos.org/plosmedicine/article?id=10.1371/journal.pmed.1003015>, we suggest the following wording:  Data Availability: Data are collected by the German Institute for Economic Research ([<https://www.diw.de/en>](http://www.kantarworldpanel.com/cl)). The authors are not legally permitted to share the data used for this study, but interested parties may contact the DIW representative Philipp Kaminsky to inquire about accessing this proprietary data ([soepmail@diw.de](mailto:soepmail@diw.de)). Further details about the data and access are provided here: <https://www.diw.de/en/diw_01.c.592731.en/soep.iab_bamf_soep_mig.2016.html#592737> |
| 2 | Please substitute "cross-sectional" for "retrospective" in the title |
|  | *Response: Done* |
| 3 | - demographic details are needed in the abstract as well as mean age. |
|  | *Response: We have added mean age and sex percentages.* |
| 4 | - p<0.01 -> exact value or p< 0.001 please |
|  | *Response: There was only one instance in the paper, which was changed to <0.001* |
| *5* | *Please change gender to sex. The terms gender and sex are not interchangeable (as discussed in* [*http://www.who.int/gender/whatisgender/en/*](http://www.who.int/gender/whatisgender/en/) *); please use the appropriate term* |
|  | Response: We changed gender into sex as requested although we are not convinced that this is more appropriate. Distinguishing between gender and sex just by looking at whether it was assessed in a binary format (sex) or not (gender) does not solve the problem. Biological sex refers to differences in chromosomes, hormones and/ or reproductive organs – and it is not binary. Surveys (like the one we used) usually ask people to self-identify as women or men. This indicator does neither measure sex directly (because they are not asked for example whether they have a XY or XX chromosome) nor gender (because they are not asked about gender roles, femininity, masculinity etc.) – it is an indicator for both. Rather than distinguishing between sex and gender based on the question format researchers should reflect whether they assume that biological or social differences between men and women lead to differential outcomes and then chose the appropriate term. In our case, we are convinced that differences in gender norms are responsible for the observed differences in SES-health satisfaction relationship. Therefore we chose gender and not sex in our original version.  If the journal agrees with this reflection, a consideration to change the journal guidelines regarding the use of sex and gender might be warranted. |
| 6 | - introduction is very long – please shorten to improve accessibility |
|  | *Response: We shortened the introduction accordingly.* |
| 7 | Did your study have a prospective protocol or analysis plan? Please state this (either way) early in the Methods section.  .  a) If a prospective analysis plan (from your funding proposal, IRB or other ethics committee submission, study protocol, or other planning document written before analyzing the data) was used in designing the study, please include the relevant prospectively written document with your revised manuscript as a Supporting Information file to be published alongside your study, and cite it in the Methods section. A legend for this file should be included at the end of your manuscript.  b) If no such document exists, please make sure that the Methods section transparently describes when analyses were planned, and when/why any data-driven changes to analyses took place.  c) In either case, changes in the analysis—including those made in response to peer review comments—should be identified as such in the Methods section of the paper, with rationale. |
|  | Response: We added this paragraph to the Methods part:  “No prospective analytical protocol was created. The main research question about the importance of social economic status for Syrian refugee health was formulated prior to data inspection and originated from the theories discussed in the introduction. The authors quickly settled on the initial difference-in-difference approach using retrospective survey information and later supplemented the core results by further robustness and sub-group analyses based on collegial feedback (i.e. Table 3) and reviewer comments (i.e. Figure A1 and Table A8). This data provides robustness checks to highlight the link between our main outcome and more objective health measures, as well as the problem of potential ceiling effects.” |
| 8 | I cannot see a table with absolute numbers of participants and their demographic information. Please provide this in the main text and ideally this should be table 1 and re-order others tables accordingly. |
|  | *Response: we hope to have updated table 1 in line with this request. We used the same format to address the reviewers request below.* |
|  | STROBE – please be accurate and provide sections instead of ‘first part’. |
|  | *Response: We provided the specific paragraphs where the respective information can be found.* |
|  | Comments from Reviewers: |
|  | Reviewer #1: The authors have addressed our original points satisfactorily; the additional clarifying analyses/charts were appreciated. |
| 9 | Given the emphasis on the 5 SES categories in the analysis (as also illustrated in Figures 1 to 4), it might be appropriate to include a breakdown of characteristics (as from Table 1) by these SES categories, including the number of subjects in each category. |
|  | *Response: We have changed table 1 in response to a request by the editor. Table A2 now provides a replication broken down by SES. We briefly comment on this in the text.* |
| 10 | The conflation of "health" and "health satisfaction" throughout the text remains slightly concerning; authors might consider re-emphasizing the use of health satisfaction from surveys as a proxy for health, at opportune points in the manuscript (e.g. the beginning of the discussion section) |
|  | *Response: We added a sentence in the second paragraph in the discussion reemphasizing that health satisfaction and self-rated health are valid predictors of objective health status.* |
|  | *Supplemental information:* |
| *11* | *Upon review of our code we found a small coding error that led to inaccurate point estimates in previous Table A8 –now A9- column 3. The correct results provide different point estimates in the Tobit Model, but the interpretation remains qualitatively unchanged.* |
